# Dietary Quality and Intake of Cancer Caregivers: A Systematic Review of Quantitative Studies and Recommendations for Future Research

**DOI:** 10.1002/cam4.70668

**Published:** 2025-02-10

**Authors:** Susannah K. Ayre, Katelyn E. Collins, Xanthia E. Bourdaniotis, Grace L. Rose, Gosia Boardman, Constantina Depaune, Belinda C. Goodwin, Elizabeth A. Johnston

**Affiliations:** ^1^ Viertel Cancer Research Centre Cancer Council Queensland Fortitude Valley Queensland Australia; ^2^ School of Exercise and Nutrition Sciences Queensland University of Technology (QUT) Kelvin Grove Queensland Australia; ^3^ School of Psychology and Wellbeing University of Southern Queensland Springfield Queensland Australia; ^4^ School of Health University of the Sunshine Coast Sippy Downs Queensland Australia; ^5^ School of Public Health and Social Work Queensland University of Technology (QUT) Kelvin Grove Queensland Australia; ^6^ Centre for Health Research University of Southern Queensland Springfield Queensland Australia; ^7^ School of Population and Global Health University of Melbourne Carlton Victoria Australia; ^8^ Population Health Program, QIMR Berghofer Medical Research Institute Herston Queensland Australia

**Keywords:** behavioral science, cancer prevention, cancer risk factors, epidemiology and prevention, nutrition

## Abstract

**Aims:**

As more people live with and beyond a cancer diagnosis, the role of informal caregivers becomes increasingly vital. Despite emotional, physical, and financial challenges, the impact of caregiving on health behaviors, including diet, has been largely overlooked. This systematic review synthesized quantitative evidence on dietary quality and intake among cancer caregivers.

**Methods:**

Five databases (CINAHL, Embase, PubMed, PsycINFO, Web of Science) were searched in February 2024 using keywords including cancer, caregiver, and diet. Articles published since 2013 that quantitatively assessed the dietary quality or intake of cancer caregivers were eligible. Articles were independently screened in Rayaan by two authors, with discrepancies resolved by a third author. Data on study design, aims, methods, sample characteristics, and results were extracted and summarized using descriptive analyses. One author performed data extraction, with a second author reviewing results for accuracy.

**Results:**

Of 12,584 records identified, 22 met eligibility criteria. Most studies were conducted in the United States (68%), were cross‐sectional (77%), and included caregivers who were partners (68%) of people with cancer. Four (18%) studies reported on energy or nutrient intakes, 13 (59%) reported on food or food group intakes, and 10 (45%) reported on diet quality or dietary patterns. Results varied widely due to differences in assessment methods used. Dietary changes due to caregiving were described in 8 (36%) studies, mostly using retrospective self‐reported data. Negative, positive, and no dietary changes were reported in 7 (32%), 5 (23%), and 4 (18%) studies, respectively. Two (9%) studies did not specify the direction of change. Eight (36%) studies assessed adherence to dietary recommendations, with mixed results.

**Conclusions:**

Evidence of the dietary quality and intake of cancer caregivers is inconclusive. Larger, longitudinal studies using validated measures, repeated observations, and comparison to dietary guidelines are needed to better understand the impacts of caregiving on diet.

## Graphical abstract

This systematic review synthesized current quantitative evidence on the dietary quality and intake of cancer caregivers. There is some evidence to suggest that caring for someone with cancer may negatively impact diet, though findings are largely inconclusive. Larger, longitudinal studies using validated measures, repeated observations, and comparison to individual or population‐based guidelines are needed to better understand the impacts of caregiving on diet.

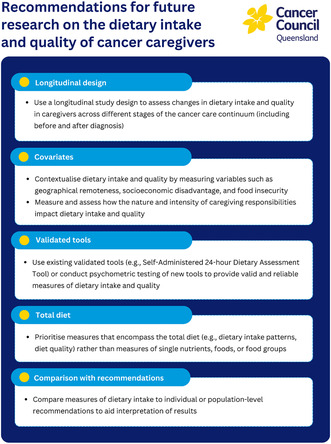

## 1 Introduction

Over recent decades, rates of cancer incidence and survival have increased worldwide [1]. With a growing number of cancer survivors, the delivery of post‐treatment support is shifting from hospital to community‐based models of care [2], resulting in an increased reliance on informal caregivers (i.e., relatives and friends) to provide support to people diagnosed with cancer. The responsibilities of caregivers can include monitoring and managing symptoms, assisting with activities of daily living (e.g., food preparation), and providing psychosocial support [3]. On average, it has been estimated that caregivers spend approximately 8 h per day providing this care (equivalent to a full‐time job) [4] and can incur more than $3500 CAD ($2600 USD) per month through direct costs, such as transport and medication, and indirect costs, such as lost income due to reduced work hours [5]. Caregiving is therefore a complex and demanding role, and has been shown to contribute to physical, psychological, and financial burden among individuals [6].

Despite the burden associated with caregiving, its responsibilities can elicit positive impacts, such as improved relationship quality and resilience among caregiver‐patient dyads [7, 8]. Following a cancer diagnosis, caregivers may also experience posttraumatic emotional growth, driven by a greater sense of purpose and appreciation for life that arises from their caregiving role [9, 10]. However, research also demonstrates the negative impacts of caregiving. For example, compared to the general population, cancer caregivers often experience poorer health outcomes [11], such as lower mental and physical quality of life [12, 13]. These outcomes can exacerbate the burden placed on caregivers [14], hindering their ability to provide support [15]. It is therefore imperative for healthcare professionals to understand and address the modifiable determinants of health within this population group.

Diet constitutes a key determinant of health, with higher diet quality associated with lower risks of cardiovascular disease, type 2 diabetes mellitus, and cancer in the general population [16]. Adhering to a healthier diet may also increase perceived quality of life [17], and protect against depression and anxiety [18]. However, in a recent qualitative study of cancer caregivers in rural Australia, it was evident that diet quality was often compromised while providing care, with participants reporting increased reliance on convenience foods and reduced meal consistency since caring for someone with cancer [19]. These changes occurred as a result of caregiver fatigue and stress, as well as reduced access to cooking facilities while away from home during treatment periods [19]. As such, diet may represent a key intervention target for improving health outcomes among cancer caregivers.

Recent evidence on the dietary quality and intake of cancer caregivers has not been comprehensively reviewed. While several qualitative studies have explored how the caregiving role may influence eating behaviors, such as perceived changes in meal frequency and food choices [19, 20, 21, 22, 23, 24, 25], quantitative studies using structured dietary assessment methods (e.g., 24‐h dietary recalls, dietary screeners) enable the dietary intake of cancer caregivers to be quantified and assessed for adequacy against individual or population‐based guidelines [26]. In 2013, Ross and colleagues [27] published a systematic review on the health behaviors of cancer caregivers, including dietary intake. Of the eight studies included in that review, three reported on dietary intake using quantitative measures; however, their results were largely conflicting and inconclusive [27]. This systematic review provides an updated synthesis of quantitative evidence from literature published in the past decade regarding dietary quality and intake among cancer caregivers. Considering that there are multiple dietary assessment methods that can influence the degree of accuracy in outcomes [26], a secondary aim of this review was to describe and evaluate the methods used to measure and interpret dietary data.

## 2 Methods

### 2.1 Protocol and Registration

The protocol for this systematic review was designed in accordance with the Joanna Briggs Institute (JBI) methodological guidance for systematic reviews [28] and registered on PROSPERO (reference number: CRD42023450937). The Preferred Reporting Items for Systematic Reviews and Meta‐Analyses (PRISMA) 2020 statement was used to report this systematic review [29].

### 2.2 Eligibility Criteria

For inclusion in this review, studies needed to quantitatively measure and report the dietary intake of cancer caregivers, including intake of energy, nutrients, foods, or food groups, or their overall diet quality or dietary intake patterns. Quantitative methods included any structured dietary assessment tool (e.g., 24‐h dietary recalls, food frequency questionnaires, dietary screeners) that measured dietary outcomes numerically (e.g., number of serves, diet quality scores, questionnaire subscale scores). Other outcomes related to, but not a direct measure of, dietary quality or intake were excluded (e.g., meal acquisition and eating behaviors). Cancer caregivers were defined as people aged ≥ 18 years who provide informal care to an adult or child diagnosed with cancer (e.g., relatives, friends). Studies that also included participants who were not cancer caregivers were eligible if the dietary quality or intake of cancer caregivers were measured and reported separately from other groups (e.g., cancer patients). For intervention trials, data on the dietary quality or intake of cancer caregivers had to be available either at baseline or from a control group to assess these outcomes in the absence of an intervention. Original full‐text studies that were published in English within the past decade (i.e., from 2013) were eligible for inclusion.

### 2.3 Information Sources and Search Strategy

The search was conducted on 15 June 2023, then updated on 2 February 2024. Five electronic databases were searched (CINAHL, Embase, PubMed, PsycINFO, and Web of Science) using a combination of key words (in title and/or abstract fields) and index terms relevant to cancer, caregiver, and diet. The search strategy was developed by the authors and adapted separately for each database (see full search syntax in Data S1). Five articles known to meet inclusion criteria [30, 31, 32, 33, 34] were used by one author to test that the search strategy retrieved relevant results. The reference lists of all included studies were screened for additional citations. Following the search, all identified citations were collated and uploaded into Rayyan [35], with duplicates removed prior to screening.

### 2.4 Study Selection

**FIGURE 1:**
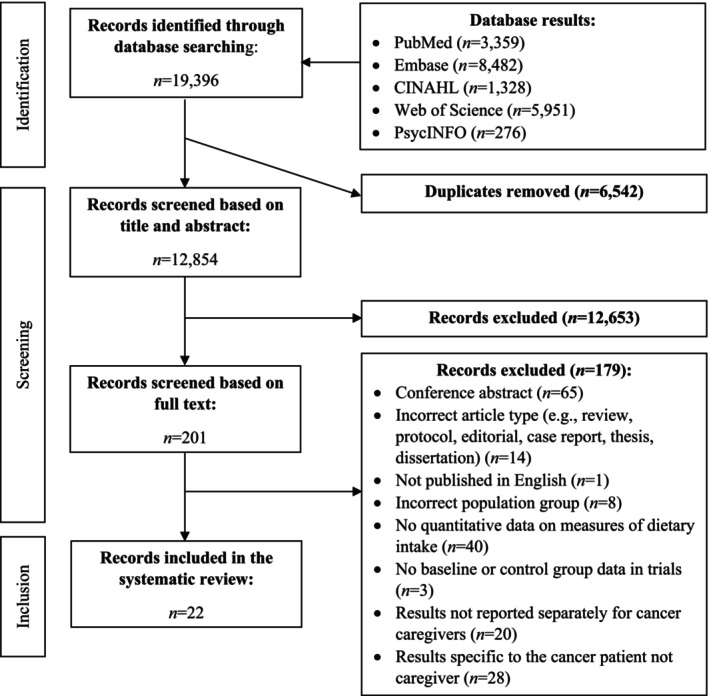
Flow chart for study selection in a systematic review on the dietary quality and intake of cancer caregivers.

Screening was performed independently by two authors. First, the titles and abstracts of all records were screened against the eligibility criteria. If eligibility could not be determined, the record proceeded to the full‐text review. Discrepancies in the full‐text review were resolved through the decision of a third author. Reasons for exclusion of the studies during the full‐text review stage were documented (see Figure 1). Full‐text studies were included if they met all eligibility criteria listed above.

### 2.5 Quality Appraisal

The quality of the included studies was assessed by one author or another research team member using the Mixed Methods Appraisal Tool (MMAT) 2018 version [36], and checked for accuracy by a second author. For the mixed‐methods studies included in this review [37, 38], only the quantitative methods were critiqued using the relevant criteria. Discrepancies were resolved through consensus after discussion with a third author. A total score was calculated for each study by summing the number of criteria met.

### 2.6 Data Extraction and Synthesis

A standardized form was used to extract relevant data from each article, including the publication details (e.g., authors, year, location), participant characteristics (e.g., relationship to patient, patient cancer type, patient status), study design (e.g., aims, study type, measurement tools), and results (e.g., energy intake, diet quality scores). Descriptive analyses (i.e., frequency counts and percentages) were used to describe the participants, design, and results of the included studies, presented in both narrative and tabular formats. For the mixed‐methods studies included in this review [37, 38], only data relevant to quantitative components of the studies were extracted. Data extraction and synthesis were performed by one author, then reviewed by a second author for completeness and accuracy. Any disagreements were resolved through consensus.

## 3 Results

### 3.1 Overview of Included Studies

After duplicates were removed, a total of 12,584 citations were identified through searches of the electronic databases. Of these citations, 22 met eligibility criteria and were included in the review [30, 31, 32, 33, 34, 37, 38, 39, 40, 41, 42, 43, 44, 45, 46, 47, 48, 49, 50, 51, 52, 53] (see Figure 1). The included articles reported on cross‐sectional (*n* = 17; 77%) [30, 34, 37, 38, 39, 41, 43, 44, 45, 46, 47, 48, 49, 50, 51, 52, 53], longitudinal (*n* = 2; 9%) [31, 32], and randomized controlled trial (RCT; *n* = 3; 14%) [33, 40, 42] studies. One (5%) study was a feasibility trial (*n* = 1; 5%) [33].

### 3.2 Characteristics of Caregivers in the Included Studies

The characteristics of the included studies are summarized in Table 1. Most studies were conducted in the United States (*n* = 15; 68%) [30, 32, 37, 40, 41, 42, 43, 44, 46, 47, 48, 49, 51, 52, 53]. In 15 (68%) studies, all or most caregivers were the intimate partner of the person with cancer [30, 31, 32, 33, 34, 37, 40, 41, 42, 43, 44, 47, 48, 51, 52], and in 3 (9%) studies, all caregivers were an adult child of the person with cancer [39, 46, 53]. Thirteen (59%) studies included caregivers of people with any cancer type [30, 31, 33, 34, 38, 40, 42, 43, 44, 47, 49, 50, 51], most of which predominantly involved caregivers of women with breast cancer (*n* = 8; 38%) [30, 31, 33, 38, 40, 42, 47, 50]. Other studies focused on a specific cancer type, for example, lung (*n* = 2; 9%) [41, 45], colorectal (*n* = 1; 5%) [32], head and neck (*n* = 1; 5%) [48], prostate (*n* = 1; 5%) [52], and gastrointestinal (*n* = 1; 5%) [37] cancer. The studies included caregivers of people at different stages of their cancer treatment, most commonly those who had completed (*n* = 6; 27%) [32, 33, 40, 45, 46, 52] or were undergoing (*n* = 5; 23%) [34, 37, 47, 49, 53] active treatment.

**TABLE 1:** Key characteristics of quantitative studies included in a systematic review on the dietary quality and intake of cancer caregivers (*n* = 22).

Study details			Caregiver baseline characteristics						Patient baseline characteristics	
First author (year)	Country	N	Age^a^ (years)	Gender (% female)	Ethnicity	Education	Employment	Relationship to patient	Cancer type	Treatment status
Ávila‐Montiel (2013) [39]	Mexico	53	33.2 (9.2)	81%	Not reported	Not reported	Not reported	Parent (87%), other (13%)	Not reported (pediatric)	Not reported
Carmack (2021) [40]	United States	22	63.4 (8.2)	41%	Non‐Hispanic White (73%), Hispanic (14%), non‐Hispanic Black (9%), other (5%)	Not reported	Not reported	Spouse	Breast (59%), prostate (36%), colorectal (5%)	Post‐treatment
Cooley (2013) [41]	United States	37	49 (18–65)^b^	70%	White (89%), Black (11%)	Not reported	Employed (65%), retired or unemployed (16%), other (14%)	Spouse or partner (57%), child (43%)	Lung	Not reported
Crane (2021) [33]	United States, Mexico	34	53 (not reported)	Not reported	Latin American	High school or below (12%), tertiary (88%)	Employed (50%), retired or not working (50%)	Spouse or partner (29%), sibling (12%), child (32%), parent (6%), friend (26%), other (3%)	Solid tumor, majority breast (68%)	Post‐treatment
Demark‐Wahnefried (2023) [42]	United States	56	Not reported	Not reported	Not reported	Not reported	Not reported	Spouse (41%), sibling (13%), child (11%), friend (30%), other (5%)	Breast (80%), other (20%)	Not reported
Dionne‐Odom (2017) [43]	United States	294	65.5 (12.7)	73%	White (91%), Black (8%), Asian (< 1%), American Indian or Native Alaskan (< 1%)	Not reported	Retried (54%), employed (23%), unemployed (9%), other (14%)	Spouse or partner (60%), parent (16%), child (11%), sibling or other family member (8%), friend (3%)	Lung (39%), head and neck (21%), blood (10%), ovarian (10%), other (20%)	Not reported
Ellis (2017) [30]	United States	484	56.5 (13.4)	57%	White (80%), Black (16%), multi‐racial (3%), Asian (1%)	14.6 (2.8)^d^	Not reported	Spouse (70%), child (15%), sibling or other family member (6%), friend (4%), unknown (5%)	Breast (32%), colorectal (29%), prostate (13%)	Undergoing treatment
Ezendam (2019) [31]	Denmark	672	57 (4.1)	55%	Not reported	10 years or less (82%), more than 10 years (18%)	Not reported	Partner	Breast (17%), prostate (11%), other (72%)	Not reported
Hecht (2021) [44]	United States	77	64 (10.7)	60%	White (97%)	High school or below (37%), tertiary (48%)	Not reported	Spouse or partner (81%), child (7%), sibling (4%), other (9%)	Liver (33%), breast (14%), colorectal (12%), lung (10%), other (31%)	Not reported
Koca (2013) [45]	Turkey	246	46 (20–83)^c^	70%	Not reported	High school or below (54%), tertiary (39%)	Not reported	Child (36%), spouse (35%), sibling (14%), parent (15%)	Lung	Not reported
Marchak (2023) [46]	United States	46	Not reported	Not reported	Not reported	Not reported	Not reported	Parent	Not reported (pediatric)	Post‐treatment
Mazanec (2015) [47]	United States	39	57.18 (not reported)	46%	White (68%), Black (32%)	High school or below (49%), tertiary (51%)	Employed (59%), unemployed (41%)	Spouse (77%), child (23%)	Breast (56%), prostate (14%), head and neck (14%), colorectal (8%), lung (8%)	Undergoing treatment
Milliron (2023) [37]	United States	27	35–54 (15%) ≥ 55 (85%)	59%	White (81%), Black (15%), Asian (4%)	High School graduate (18%), Some college (15%), College graduate (41%), Graduate school (26%)	Full‐time paid work (19%), Part‐time paid work (7%), Homemaker (7%), Unemployed (7%), Receiving disability (4%), Retired (52%), Other (4%)	Spouse (93%), parent or child (7%)	Gastrointestinal	Undergoing treatment
Nightingale (2016) [48]	United States	33	60 (11.2)	82%	Non‐Hispanic White (85%), non‐Hispanic Black (12%), Asian (3%)	High school or below (52%)	Employed (39%)	Spouse or partner (73%), parent (12%), child (3%), other (12%)	Head and neck	Pre‐treatment, undergoing treatment, or post‐treatment
Packel (2023) [49]	United States	52	Not reported	81%	White (88%), Black (4%), other (8%)	High school or below (12%), tertiary (88%)	Employed (34%), not working (64%)	Not reported	Hematological (21%), head, neck, or lung (21%), gastrointestinal (17%), other solid tumors (42%)	Undergoing treatment
Piazza (2017) [38]	Italy	93	Not reported	Not reported	Not reported	Not reported	Not reported	Not reported	Breast (32%), gastrointestinal (15%), other (53%)	Pre‐treatment, undergoing treatment, post‐treatment, or undergoing palliative care
Rha (2015) [34]	South Korea	227	46.6 (11.98)	81%	Not reported	High school or below (49%), tertiary (51%)	Employed (35%), unemployed (65%)	Spouse (49%), child (35%), other family member (15%)	Lung (21%), colorectal (20%), gastrointestinal (16%), breast (11%), other (33%)	Undergoing treatment
Rillamas‐Sun (2022) [50]	United States (72%), other (28%)	266	18–35 (10%) 36–55 (37%) 56–65 (27%) 66–75 (21%) 76+ (4%)	88%	White (70%), mixed‐racial (13%), Asian, Native Hawaiian, or Pacific Islander (6%), Black (4%), other (7%)	High school or below (10%), tertiary (90%)	Not reported	Not reported	Breast (44%), gastrointestinal (15%), other (41%)	Not reported
Ross (2020) [51]	United States	129	48.6 (11.8)	67%	White (71%), Hispanic or Latin American (15%), Black (14%)	High school or below (5%), tertiary (95%)	Employed (74%)	Spouse (50%), parent (35%), other (15%)	Skin (20%), hematological (14%), other (66%)	Pre‐treatment or undergoing treatment
Shaffer (2016) [32]	United States	162	56 (13.33)	80%	White (53%), Black (41%), other (6%)	High school or below (29%), tertiary (61%)	Not reported	Spouse or partner (52%), child or child‐in‐law (20%), other (28%)	Colorectal	Not reported
Virtue (2015) [52]	United States	66	57.1 (8.8)	Not reported	White (76%), Black (16%), Hispanic or Latin American (5%), Asian (2%), other (1%)	High school or below (14%), tertiary (86%)	Employed (67%), not working (33%)	Partner	Prostate	Post‐treatment
Wiener (2016) [53]	United States	263	Not reported	82%	White (67%), Black (14%), mixed‐racial (3%), Asian (2%), Native Hawaiian or Alaskan (1%), other (7%)	High school or below (32%), tertiary (58%)	Not reported	Parent	Not reported	Undergoing treatment

^a^ Sample mean (standard deviation), unless otherwise specified.

^b^ Sample median (range).

^c^ Sample mean (range).

^d^ Highest level of education in years, sample mean (standard deviation).

Across the 17 studies that reported on the gender of caregivers, the proportion of females ranged from 41% to 88% [30, 31, 32, 34, 37, 39, 40, 41, 43, 44, 45, 47, 48, 49, 50, 51, 53]. Two (9%) studies reported on the geographical remoteness of the sample, with caregivers in one study mostly residing in urban areas [50] and caregivers in the other study mostly residing in rural areas [43]. Seventeen (77%) studies reported on at least one indicator of socioeconomic status, including educational attainment, employment status, and income [30, 31, 32, 33, 34, 37, 41, 43, 44, 45, 47, 48, 49, 50, 51, 52, 53]. Most caregivers in these studies had received at least a high school level education and were currently employed. While the reporting of average incomes varied, four (18%) studies assessed the perceived adequacy of financial resources or support among caregivers [37, 44, 48, 49]. In these studies, most caregivers reported receiving adequate financial resources or support. In the 1 (5%) study that measured food security, most caregivers reported experiencing high levels of food security [33].

A total of 10 (45%) studies reported on relationship status, with all or most caregivers in these studies either married or partnered [30, 33, 37, 41, 43, 44, 47, 51, 52, 53]. In addition, 15 (68%) studies reported on race or ethnicity, with most caregivers identifying as White [30, 32, 33, 37, 40, 41, 43, 44, 47, 48, 49, 50, 51, 52, 53]. Other caregiver characteristics described included weight status [31, 33, 39, 40, 44] and the presence of comorbidities [33, 37, 41, 45, 50]. Some studies reported details about the caregiver's role, including caregiver burden [34, 51], the duration [43, 51] and frequency [37, 43, 48, 49, 51] of providing care, division of caring responsibilities [34, 51], and living arrangements with the person diagnosed with cancer [30, 34, 45, 47, 51, 52].

### 3.3 Quality Appraisal

Most studies (*n* = 14, 64%) included in this review met at least three of the five quality criteria on the MMAT [31, 32, 34, 37, 39, 40, 41, 42, 43, 46, 47, 48, 50, 52] (see Table 2). The most common unmet criterion was for low risk of non‐response bias (*n* = 13; 59%), typically resulting from high non‐response rates and limited reporting of reasons for non‐responses. Representativeness of the target population within the sample was also a common unmet criterion (*n* = 10; 45%), often due to a reliance on convenience sampling and small sample sizes, resulting in the under‐representation of specific demographic or clinical subgroups. Similarly, use of appropriate measurements was a common unmet criterion (*n* = 9; 41%) due to the lack of reporting on instrument validity and reliability estimates.

**TABLE 2:**
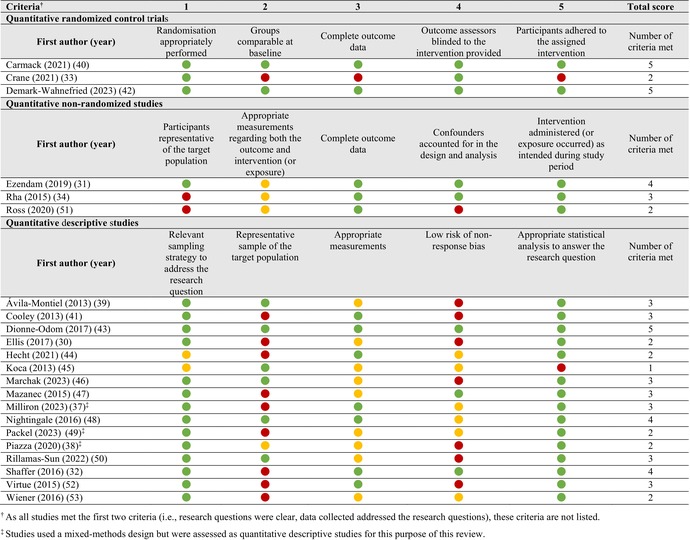
Quality assessment of quantitative studies included in a systematic review on the dietary quality and intake of cancer caregivers using the 2018 Mixed Methods Appraisal Tool (MMAT) (*n* = 22).

*Note*: Green = criterion met; Red = criterion not met; Yellow = unclear.

### 3.4 Dietary Quality and Intake Among Cancer Caregivers

Measures of cancer caregiver dietary quality and intake were categorized across three broad domains: (i) energy and nutrient intakes, (ii) food and food group intakes (including fruit and vegetables, discretionary foods and drinks, meats and alternatives, dairy products, and unsaturated oils), and (iii) diet quality and dietary intake patterns. Measures and outcomes are reported below and summarized in Tables 3, 4, and 5, respectively for each domain.

**TABLE 3:** Results from quantitative studies reporting on energy and/or nutrient intakes in cancer caregivers (*n* = 4).

		Intake (frequency or amount)		
First author (year)	Measurement	Mean (standard deviation)		
Ávila‐Montiel (2013) [39]	Method: 1 × 24‐h dietary recall Tool: 24‐h Food Reminder (R24) Timepoints: T1: Baseline (*n* = 53) T2: 24 h post‐baseline (*n* = 50) T3: 48 h post‐baseline (*n* = 40)	**Calories (kcal/day)** T1: 1534.0 (1393.8) T2^a^: 1469.1 (1276.6) T3^a^: 1901.0 (1972.0) Protein (g/day) T1: 46.0 (28.0) T2^a^: 44.0 (21.0) T3^a^: 52.5 (28.8)	**Fat (g/day)** T1: 44.0 (30.2) T2^a^: 41.0 (28.0) T3^a^: 54.9 (65.0) **Carbohydrates (g/day)** T1: 240.2 (329.0) T2^a^: 232.0 (304.0) T3^a^: 302.0 (441.0)	**Dietary fiber (g/day)** T1: 12.1 (11.0) T2^a^: 8.0 (5.0) T3^a^: 11.2 (11.0)
Carmack (2021) [40]	Method: 2 × 24‐h dietary recalls (results averaged) Tool: Automated Self‐Administered 24‐h Dietary Assessment Tool (ASA24) [54] Timepoints (control group): T1: Baseline (*n* = 9) T2: 6‐month follow‐up (*n* = 8) Timepoints (intervention group): T1: Baseline (*n* = 12)	**Fat (g/day)** T1 (control group): 58.9 (26.3) T2 (control group)^b^: 51.1 (27.3) T1 (intervention group): 85.9 (38.8)	**Saturated fat (g/day)** T1 (control group): 20.1 (10.1) T2 (control group)^b^: 18.0 (10.9) T1 (intervention group): 28.5 (13.0)	
Crane (2021) [33]	Method: 30‐day dietary screener Tool: National Cancer Institute (NCI) Dietary Screener Questionnaire (DSQ)—English and Spanish versions (19 items) [55, 56] Timepoints (control group): T1: Baseline (*n* = 13) T2: 12‐week follow‐up (*n* = 9) Timepoints (intervention group): T1: Baseline (*n* = 21)	**Dietary fiber (g/day)** T1 (control group): 16.6 (3.1) T2 (control group)^a^: 17.3 (1.2) T1 (intervention group): 16.7 (4.6)		
Demark‐Wahnefried (2023) [42]	Method: 2 × 24‐h dietary recalls (results averaged) Tool: ASA24 [54] Timepoints (control group): T1: Baseline (*n* = 28) T2: 6‐month follow‐up (*n* = 24) Timepoint (intervention group): T1: Baseline (*n* = 28)	**Calories (kcal/day)** T1 (control group): 1553.5 (483.7) T2 (control group)^c^: 1467.2 (501.3) T1 (intervention group): 1570.2 (498.3)		

		Intake (adherence to dietary recommendations)
First author (year)	Measurement	Mean (standard deviation)
Ávila‐Montiel (2013) [39]	Method: 1 × 24‐h dietary recall Tool: 24‐h Food Reminder (R24) Recommendations: World Health Organization (WHO) formula Timepoints: T1: Baseline (*n* = 53) T2: 24 h post‐baseline (*n* = 50) T3: 48 h post‐baseline (*n* = 40)	**Calories (% of recommended intake)** T1: 66.3 (56.0) T2^a^: 64.0 (54.0) T3^a^: 82.0 (78.0)

^a^ Comparison with previous timepoint not performed.

^b^ Comparison with previous timepoint performed but not statistically significant.

^c^ Comparison with previous timepoint performed and statistically significant.

**TABLE 4:** Results from quantitative studies reporting on food or food group intakes in cancer caregivers (*n* = 13).

First author (year)	Measurement	Intake (frequency or amount)	
		***Mean (standard deviation)***	
Carmack (2021) [40]	Method: 2 × 24‐h dietary recalls (results averaged) Tool: Automated Self‐Administered 24‐h Dietary Assessment Tool (ASA24) [54] Timepoints (control group): T1: Baseline (*n* = 9) T2: 6‐month follow‐up (*n* = 8) Timepoints (intervention group): T1: Baseline (*n* = 12)	**Fruits and vegetables (cups/day)** T1 (control group): 2.8 (1.5) T2 (control group)^a^: 3.3 (1.3) T1 (intervention group): 2.4 (1.3)	
Crane (2021) [33]	Method: 30‐day dietary screener Tool: National Cancer Institute (NCI) Dietary Screener Questionnaire (DSQ)—English and Spanish versions (19 items) [55, 56] Timepoints (control group)^b^: T1: Baseline (*n* = 13) T2: 12‐week follow‐up (*n* = 9) Timepoints (intervention group)^b^: T1: Baseline (*n* = 21)	**Fruits and vegetables (cups/day)** T1 (control group): 2.7 (1.1) T2 (control group)^c^: 2.6 (0.2) T1 (intervention group): 2.5 (0.8) **Fruits (cups/day)** T1 (control group): 1.2 (0.9) T2 (control group)^c^: 0.9 (0.1) T1 (intervention group): 0.9 (0.4) Vegetables (cups/day) T1 (control group): 1.6 (0.5) T2 (control group)^c^: 1.6 (0.1) T1 (intervention group): 1.6 (0.5)	**Added sugar (g/day)** T1 (control group): 16.2 (4.8) T2 (control group)^c^: 13.8 (0.7) T1 (intervention group): 15.7 (5.7) **Sugar from sugar‐sweetened beverages (g/day)** T1 (control group): 7.4 (4.2) T2 (control group)^c^: 5.7 (0.4) T1 (intervention group): 7.1 (4.8)
Ezendam (2019) [31]	Method: Dietary screener Tool: Study specific Timepoints: T1: Baseline (before cancer diagnosis; *n* = 672) T2: 3‐9‐year follow‐up (after cancer diagnosis; *n* = 672)^d^	**Fruits (pieces/day)** T1: 1.1 (1.0) T2^c^: 1.7 (1.5)	**Sugar‐sweetened beverages (ml/day)** T1: 86 (170) T2^c^: 53 (131)
Hecht (2021) [44]	Method: Dietary screener Tool: Dietary Behavior and Nutrition Questionnaire (DBQ) [57] Timepoint: Single timepoint (*n* = 78). Data on fruit and vegetable intake were available for 67 and 69 caregivers, respectively.	**Fruits (serves/day)** 1.26 (1.04)	**Vegetables (serves/day)** 1.75 (1.21)
Nightingale (2016) [48]	Method: Dietary screener Tool: NCI Food Attitudes and Behaviors (FAB) Survey [58, 59] Timepoint: Single timepoint (*n* = 33)	**Fruits and vegetables (cups/day)** 2.7 (1.7)	
Packel (2023) [49]	Method: Dietary screener Tool: NCI FAB Survey [59, 67] Timepoint: Single timepoint (*n* = 52)	**Fast food (meals/week)** 1	
		***Proportion (%) of sample***	
Packel (2023) [49]	Method: Dietary screener (previous month) Tool: NCI FAB Survey [58, 59] Timepoint: Single timepoint (*n* = 52)	**Fruits and vegetables** > 1 time/week—53% 1 time/week—17% 1–2 times/month—20% < 1 time/month—10%	
Rillamas‐Sun (2022) [50]	Method: Dietary screener Tool: Study specific Timepoint: Single timepoint (*n* = 266). The amount of fruit and vegetable intake was measured in subsamples of caregivers who reported consuming these foods (*n* = 224 and *n* = 225, respectively).	Fruits Never—3% 1–3 days/week—16% 4–6 days/week—30% Daily—30% **Fruits** < 1 cup—60% 1–2 cups—29% > 2 cups—11%	**Vegetables** Never—0% 1–3 days/week—11% 4–6 days/week—32% Daily—44% **Vegetables** < 1 cup—52% 1–2 cups—35% > 2 cups—12%

First author (year)	Measurement	Intake (adherence to dietary recommendations)	
		***Proportion (%) of sample***	
Cooley (2013) [41]	Method: Dietary screener Tool: Previously used item—i.e., ‘How many days per week do you eat at least five servings of fruits and vegetables?’ [60] Recommendations: American Cancer Society (ACS) Timepoint: Single timepoint (*n* = 37)	**Fruits and vegetables** Meeting recommendations (≥ 5 serves/day)—5%	
Piazza (2017) [38]	Method: Structured interview questions Tool: Study specific Recommendations: World Health Organization (WHO) Timepoint: Single timepoint (*n* = 132)	**Fruits and vegetables** Low consumption (< 4 portions/day)—82% **Meat** High consumption (> 3 portions/week)—80%	**Fish** High consumption (> 2 portions/week)—70% Sugary drinks Low consumption (< 1 portion/day)—70%
Virtue (2015) [52]	Method: Dietary screener (previous week) Tool: Validated tool [61] Recommendations: ACS Timepoint: Single timepoint (*n* = 66)	**Fruits and vegetables** Meeting recommendations (≥ 5 serves/day)—64%	
		***Mean (standard deviation)***	
Shaffer (2016) [32]	Method: Dietary screener Tool: Previously used item—i.e., ‘How many days per week do you eat at least five servings of fruits and vegetables?’ [60] Recommendations: ACS (≥ 5 serves/day) Timepoints: T1: Baseline (2 months post‐diagnosis; *n* = 82) T2: 6 months post‐diagnosis (*n* = 74) T3: 12 months post‐diagnosis (*n* = 73)	**Fruits and vegetables (numbers of days/week)** T1: 2.66 (2.11) T2^e^: 2.78 (1.99) T3^e^: 3.18 (2.12)	

First author (year)	Measurement	Intake (changes since starting caregiver role)	
		***Proportion (%) of sample***	
Ezendam (2019) [31]	Method: Dietary screener Tool: Study specific Timepoints: T1: Baseline (before cancer diagnosis; *n* = 672) T2: 3‐9‐year follow‐up (after cancer diagnosis; *n* = 672)^d^	**Increased intake (≥ 25%):** Fruits—19% Sugar‐sweetened beverages—24% **Decreased intake (≥ 25%)** Fruits—51% Sugar‐sweetened beverages—48%	**No change:** Fruits—31% Sugar‐sweetened beverages—28%
Packel (2023) [49]	Method: Behavioral questionnaire Tool: Study specific Timepoint: Single timepoint (*n* = 52)	**Increased intake:** Fruits and vegetables—73% Fast food—6% **Decreased intake:** Fruits and vegetables—4% Fast food—13%	**No change:** Fruits and vegetables—24% Fast food—81%
Koca (2013) [45]	Method: Behavioral questionnaire Tool: Study specific Timepoint: Single timepoint (*n* = 246)	**Increased intake:** Fruits and vegetables—11% Fresh fruit juice—3% Milk and dairy products—6% White meat—3% Olive oil—3% Honey—5%	**Decreased intake:** Red meat—4% Fried food—5% Salty foods—2% Spices—3%
Marchak (2023) [46]	Method: Behavioral questionnaire Tool: Study specific Timepoint: Single timepoint (*n* = 46)	**Increased intake:** Unhealthy foods or snacks—15%	

^a^ Comparison with previous timepoint performed but not statistically significant.

^b^ Eligibility criteria for caregivers to participate in the study included fruit and vegetable intake of < 7 serves/day for women or < 9 serves/day for men.

^c^ Comparison with previous timepoint not performed.

^d^ Changes in fruit and sugar‐sweetened beverage intake over time were compared between individuals whose partner received a cancer diagnosis (*n* = 672) and individuals whose partner did not receive a cancer diagnosis (*n* = 5534) during the study period; however, differences were non‐significant.

^e^ Comparison with previous timepoint performed and statistically significant.

**TABLE 5:** Results from quantitative studies reporting on diet quality and dietary intake patterns or dietary intake patterns in cancer caregivers (*n* = 10).

First author (year)	Measurement	Diet quality scores	
		***Mean (standard deviation)***	
Demark‐Wahnefried (2023) [42]	Method: 2 × 24‐h dietary recalls (results averaged) Tool: Automated Self‐Administered 24‐h Dietary Assessment Tool (ASA24) [54] Recommendations: Dietary Guidelines for Americans (DGAs) Scoring metric: Healthy Eating Index‐2015 (HEI‐2015). Overall scores ranged from 0 to 100, with higher scores indicating better diet quality. Timepoints (control group): T1: Baseline (*n* = 28) T2: 6‐month follow‐up (*n* = 24) Timepoints (intervention group): T1: Baseline (*n* = 28)	**Overall diet** T1 (control group): 55.5 (10.5) T2 (control group)^a^: 54.6 (12.2) T1 (intervention group): 52.2 (12.0)	
		***Median (range), corresponding letter grade***	
Milliron (2023) [37]	Method: 3 × 24‐h dietary recalls (results averaged) Tool: ASA24 [54] Recommendations: DGAs Scoring metric: Healthy Eating Index‐2020 (HEI‐2020). Overall scores range from 0 to 100, with higher scores indicating better diet quality. Nutrient and food group scores were calculated as percentages of the maximum possible score for each nutrient or food group according to guidelines. Grades were assigned to the percentage scores as follows: ‘A’ (90%–100%), ‘B’ (80%–89%), ‘C’ (70%–79%), ‘D’ (60%–69%), and ‘F’ (0%–59%), with higher scores indicating closer alignment with recommendations. Timepoint: Single timepoint (*n =* 27)	**Overall diet** 42.0 (33.0–73.0) Unsaturated fat 0% (0%–100%), F **Saturated fat** 60% (0%–100%), D **Sodium** 40% (0%–100%), F **Fruits** Total: 20% (0%–80%), F Whole: 20% (0%–100%), F **Vegetables** Total: 40% (0%–80%), F Greens and beans: 40% (0%–100%), F	**Dairy products** 50% (10%–100%), F **Protein foods** Total: 80% (40%–100%), B Seafood and plant‐based foods: 60% (0%–100%), D **Whole grains** 20% (0%–100%), F Refined grains 70% (0%–100%), C **Added sugar** 70% (0%–100%), C
		***Proportion (%) of sample***	
Rha (2015) [34]	Method: Behavioral questionnaire (previous 30 days) Tool: Self‐Dietary Assessment Index (SDAI) [63] Recommendations: National Cancer Center of Korea Measurement scale: 3‐point Likert scale. Total scores range from 20 to 100, with scores 71–100 indicating a healthy eating pattern, 60–70 indicating a need for improvement, and < 59 indicating dietary problems. Timepoint: Single timepoint (*n* = 227)	**Overall diet** Scores 71–100—39% Scores 60–70—47% Scores < 59%–10%	

First author (year)	Measurement	Intake (adherence to healthy dietary pattern)
		***Mean (standard deviation)***
Dionne‐Odom (2017) [43]	Method: Behavioral questionnaire Tool: Health‐Promoting Lifestyle Profile‐II (HPLP‐II)—nutrition subscale [64] Measurement scale: 4‐point Likert scale, with higher scores indicating more frequent adherence to the DGAs (i.e., 1 = ‘never’ to 4 = ‘routinely’). Items were averaged to determine a mean subscale score. Timepoint: Single timepoint (*n* = 294)	2.5 (0.6)
Ross (2020) [51]	Method: Behavioral questionnaire Tool: HPLP‐II—nutrition subscale [64] Measurement scale: 4‐point Likert scale, with higher scores indicating more frequent adherence to the DGAs (i.e., 1 = ‘never’ to 4 = ‘routinely’). Items were averaged to determine a mean subscale score. Timepoint: Single timepoint (*n* = 129)	2.8 (0.5)
		***Proportion (%) of sample***
Ellis (2017) [30]	Method: Dietary screener Tool: Study specific—i.e., ‘How often do you eat a balanced diet including fruits and vegetables?’ Measurement scale: 5‐point Likert scale, with higher scores indicating more frequent intake of a balanced diet (i.e., 1 = ‘not at all’ to 5 = ‘5–7 times per week’). Timepoints (control and intervention groups):^b^ T1: Baseline (*n* = 484)	**Consuming balanced diet:** T1: ≥ 3 days/week—72%
Koca (2013) [45]	Method: Behavioral questionnaire Tool: Study specific Timepoint: Single timepoint (*n* = 246)	**Consuming diet rich in:** Fruits and vegetables—57% Protein foods—13% Grains—2% Vegetables and protein foods—14% Vegetables and grains—5% Vegetables, protein foods, and grains—5% Vegetables, grains, and fat—5%
Mazanec (2015) [47]	Method: Dietary screener (previous month) Tool: Study specific—e.g., ‘In the past month, how often did you eat a healthy diet that included a variety of recommended foods such as whole grains, fruits, vegetables, protein, and dairy?’ Timepoint: Single timepoint (*n* = 39)	**Eating healthy foods prior to diagnosis:** Usually—82% Rarely—15% Never—3% **Eating healthy foods in the past month:** Usually—77% Rarely—23% Never—0%

First author (year)	Measurement	Intake (changes since starting caregiver role)	
		***Proportion (%) of sample***	
Ávila‐Montiel (2013) [39]	Method: Behavioral questionnaire Tool: Study specific Timepoints: T1: Baseline (*n* = 53)	**Change in usual diet (during patient's hospital stay):** 82%	
Koca (2013) [45]	Method: Behavioral questionnaire Tool: Study specific Timepoints: Single timepoint measure (*n* = 246)	Change in eating habits: 14%	
Rha (2015) [34]	Method: Behavioral questionnaire Tool: Study specific Timepoint: Single timepoint (*n* = 227)	**Change in diet:** Positive change – 36% Negative change—37% No change—26%	
Ross (2020) [51]	Method: Behavioral questionnaire Tool: Study specific—i.e., ‘Since becoming a caregiver, my diet is worse’ Timepoint: Single timepoint (*n* = 129)	Worsened diet: Agree or strongly agree—47% Neither agree nor disagree—18% Disagree—21%	
Wiener (2016) [53]	Method: Behavioral questionnaire Tool: Study specific—i.e., ‘Since my child's diagnosis, my diet/nutrition has been…’ Timepoint: Single timepoint (*n* = 263)	Change in diet/nutrition: Total sample: Less healthy—62% Partnered parents: Healthier—9% Less healthy—61% Stayed the same—30%	Single parents: Healthier—8% Less healthy—64% Stayed the same—28%

^a^ Comparison with previous timepoint performed but not statistically significant.

^b^ In this study, a secondary analysis of data from a randomized controlled trial (RCT) was performed. Data collected at subsequent timepoints were not reported in this table as estimates from the intervention and control groups were combined.

#### 3.4.1 Energy and Nutrient Intake

Four (18%) studies reported on the energy and/or nutrient intakes of cancer caregivers [33, 39, 40, 42] (see Table 3). In one of two studies that measured energy intake, caregivers' diets were assessed in a hospital setting over three consecutive days using the 24‐h Food Reminder (R24) [39]. This tool was administered by a dietitian using prompts and visual aids [39]. In the second study [42], intake was assessed by a dietitian on a non‐consecutive weekday and weekend day using the Automated Self‐Administered 24‐h Dietary Assessment Tool (ASA24) [54], with results from the two assessments averaged [42]. In both studies, energy intake was reported as an average total number of calories consumed per day [39, 42]. Ávila‐Montiel et al. [39] additionally reported energy intake as an average percentage of the recommended number of calories consumed per day. Recommended intakes were determined using a World Health Organization (WHO) formula that considered the weight, height, sex, and physical activity level of individuals [39]. Across the three days, caregivers consumed an average of 64%–82% of their recommended daily caloric intake (total *n* = 40–53) [39]. For both studies, absolute intakes are reported in Table 3.

Three (14%) studies reported on the nutrient intake of cancer caregivers [33, 39, 40]. Individual nutrients included fat (total and/or saturated) [39, 40], protein [39], carbohydrates [39], and dietary fiber [33, 39]. No studies measured intake of micronutrients. Carmack et al. [40] used the ASA24 [54] to assess nutrient intake among caregivers over a single weekday and weekend day, with results from the two days averaged. Crane et al. [33] assessed how frequently caregivers consumed select foods and drinks over the previous 30 days using the Dietary Screening Questionnaire (DSQ) [55, 56], while Ávila‐Montiel et al. [39] used the R24, administered by a dietitian, to measure dietary intake in caregivers separately over three consecutive days. Absolute intakes reported across the three studies are provided in Table 3.

#### 3.4.2 Food and Food Group Intake

Thirteen (59%) studies reported on intake of foods and/or food groups in cancer caregivers [31, 32, 33, 38, 40, 41, 44, 45, 46, 48, 49, 50, 52] (see Table 4). Measures can be categorized as fruits and/or vegetables [31, 32, 33, 38, 40, 41, 44, 45, 48, 49, 50, 52], discretionary foods and/or drinks such as sugar‐sweetened beverages [31, 38, 45, 46, 49], meats and/or alternatives [38, 45], whole grains [45], dairy products [45], and unsaturated oils [45]. Many studies used existing tools to assess intake, including the ASA24 [40, 54], Food Attitudes and Behaviors (FAB) Survey [48, 49, 58, 59], DSQ [33, 55, 56], Dietary Behavior and Nutrition Questionnaire (DBQ) [44, 57], and other previously validated or used dietary screeners [32, 41, 52, 60, 61]. However, intake was also commonly assessed using study‐specific questionnaires that were often not explicitly reported [31, 38, 45, 46, 49, 50].

#### 3.4.3 Fruits and Vegetables

Twelve (55%) studies reported on fruit and/or vegetable intakes [31, 32, 33, 38, 40, 41, 44, 45, 48, 49, 50, 52]. Intake of these food groups was reported separately [31, 33, 44, 48, 50] or as a single combined measure [32, 33, 38, 40, 41, 45, 48, 49, 52]. The units of measurement varied between studies, with examples including the number of cups [33, 40, 48], serves [44], and pieces [31] consumed per day. One study compared average fruit and vegetable intake to recommendations from the WHO [44], and another study to recommendations from the National Cancer Institute (NCI) [48]. In both studies, average fruit and vegetable intake did not meet the recommendations (*n* = 33–69) [44, 48]. Three studies reported the proportion of caregivers meeting dietary recommendations for fruit and vegetable intake, according to recommendations by the WHO [38] or ACS [41, 52], with 5%–64% of caregivers meeting these recommendations (total *n* = 37–132). Another study assessed dietary intake in caregivers two months after the patient's diagnosis and found that caregivers met the ACS recommendations for fruit and vegetable intake (5 serves/day) an average of 2.7 days per week (*n* = 82) [32]. This number increased to 3.2 days per week 12 months following diagnosis (11% attrition) [32].

In the remaining studies, fruit and vegetable intake was reported more generally and without explicit reference to dietary recommendations [31, 33, 40, 45, 49, 50]. For example, Packel et al. [49] found that 53% of caregivers reported consuming fruits and vegetables on more than one occasion per week, with 73% increasing in their consumption post patient‐diagnosis (total *n* = 52). While this study suggests that fruit and vegetable intake may increase following a loved one being diagnosed with cancer, Ezendam et al. [31] measured fruit consumption in 627 people over time, and found that changes in fruit consumption did not significantly differ between people whose partner did and did not receive a cancer diagnosis during the study period. Additionally, Koca et al. [45] found that only 11% of caregivers reported an increase in their consumption of fruit and vegetables following the patient's diagnosis (total *n* = 246). Intakes reported across the other studies are provided in Table 4.

#### 3.4.4 Discretionary Foods and Drinks

Six (27%) studies reported on intake of discretionary (i.e., energy‐dense, nutrient‐poor) foods and/or drinks including food components such as added sugar [31, 33, 38, 45, 46, 49], with one of these studies referring to dietary recommendations. This study found that 70% of caregivers met recommendations by the WHO, consuming less than one serve of sugar‐sweetened beverages per day (total *n* = 132) [38]. In a cohort study comparing people whose partners were or were not diagnosed with cancer during the study period, there was no significant difference in changes to sugar‐sweetened beverage intake over time between the two groups (*n* = 672) [31]. Similarly, based on retrospective reports, Packel et al. [49] found that 81% of caregivers did not change their intake of fast food following the patient's diagnosis (total *n* = 52). In that study, 6% of caregivers reported an increase in their intake of fast food, compared to 15% of caregivers in the study by Marchak et al. [46] who reported an increase in their intake of unhealthy foods (total *n* = 46). Intake of discretionary food items reported in the remaining studies is presented in Table 4.

#### 3.4.5 Meats and Alternatives

Two (9%) studies reported on intake of meats and/or alternatives [38, 45], with one of these studies comparing data to recommendations [38]. For example, Piazza et al. [38] found that 70% of caregivers consumed an adequate amount of fish (at least two serves per week) as recommended by the WHO; however, 80% also had high meat consumption (more than three serves per week; total *n* = 132). Additionally, Koca et al. [45] found that 3% of caregivers in their sample reported increasing their consumption of white meat following the patient's diagnosis, and 4% reported decreasing their consumption of red meat (total *n* = 246).

#### 3.4.6 Milk and Dairy Products

One (5%) study measured intake of milk and dairy products. Based on retrospective reports from 246 caregivers, this study found that 6% of caregivers reported increasing their consumption of dairy products following the patient's diagnosis [45]. The percentage of caregivers who reduced or maintained their intake of milk and dairy products was not reported.

#### 3.4.7 Unsaturated Oils

Intake of unsaturated oils was measured by one study [45], which found that 3% of caregivers retrospectively reported increasing their intake of olive oil following the patient's diagnosis (*n* = 246). The percentage of caregivers who either reduced or maintained their olive oil intake was not provided.

### 3.5 Diet Quality and Dietary Intake Patterns

Ten (45%) studies reported on diet quality or dietary intake patterns in cancer caregivers [30, 34, 37, 39, 42, 43, 45, 47, 51, 53] (see Table 5). Outcomes were reported as diet quality scores [34, 37, 42], level of adherence to dietary recommendations or patterns [30, 43, 45, 47, 51], and the proportion of participants who reported changes in their diet since becoming a caregiver [34, 39, 45, 51, 53]. Demark‐Wahnefried et al. [42] and Milliron et al. [37] calculated diet quality scores using the 2015 and 2020 iterations of the Healthy Eating Index (HEI), respectively, which are diet quality indexes used to assess how closely dietary intakes align with the Dietary Guidelines for Americans (DGAs) [62]. In both studies, diet quality scores were estimated based on average intake recorded over 2–3 days using the ASA24 [54], with a dietitian administrating this tool in one of the studies [42]. On a scale of 0–100, where higher scores indicate better diet quality, typical scores ranged from 42 to 56 (*n* = 27–28) [37, 42]. Milliron et al. [37] additionally reported scores for individual nutrients, foods, and food groups. These scores were reported as a percentage of the maximum possible score for that dietary component, alongside a corresponding grade (‘A’ to ‘F’, where ‘F’ represents the lowest score) [37]. The median scores for intake of nutrients (unsaturated fat, saturated fat, and sodium) corresponded to grades within the range of ‘D’ (60%) to ‘F’ (0%), and the median scores for intake of foods and food groups (fruit, vegetables, dairy products, protein foods, whole grains, refined grains, and added sugar) corresponded to grades within the range of ‘B’ (80%) to ‘F’ (20%) [37]. In a separate study [34], adherence to a healthy eating pattern over the previous 30 days was scored using the Self‐Dietary Assessment Index (SDAI), a questionnaire adapted from the Mini Dietary Assessment Index (MDAI), based on standards from the National Cancer Center of Korea [63]. In this study, a large proportion (47%) of caregivers' scores were between 60 and 70 (out of 100), indicating a “need for improvement” in their dietary intake (total *n* = 277) [34]. Caregiver burden was not associated with participant scores in this study after adjusting for demographic and health characteristics [34].

Dietary intake patterns, including changes as a result of caregiving, were assessed cross‐sectionally, often using study‐specific questionnaires that comprised single items [30, 34, 39, 43, 45, 47, 51, 53]. For example, in two studies, most caregivers (72%–77%) reported frequently consuming a “balanced” or “healthy” diet (total *n* = 39–484) [30, 47]. In one of these studies, this percentage was slightly higher (82%) for diet prior to diagnosis, based on retrospective recall [47]. In another study, most (57%) caregivers reported consuming a diet “rich” in fruits and vegetables; however, the proportion of those adhering to other dietary patterns (e.g., diet “rich” in grains) were lower [45]. Two other studies used the nutrition subscale of the Health‐Promoting Lifestyle Profile‐II (HPLP‐II) [64] to measure how frequently caregivers adhered to recommendations in the DGAs [43, 51]. On a scale from 1 (‘never’) to 4 (‘routinely’), mean sample scores in these studies ranged from 2.5 to 2.8 (*n =* 129–294) [43, 51].

Changes in dietary intake patterns were observed in five studies [34, 39, 45, 51, 53]. In one study of 53 caregivers, 82% reported dietary changes during the patient's hospitalization [39], and in another study of 246 caregivers, 14% reported dietary changes following the patient's cancer diagnosis [45]. However, the direction of these changes was not described in either study. In three other studies, at least one quarter of participants (26%–62%) reported that caregiving had negatively impacted their diet, with the remaining caregivers reporting no changes or positive changes to their diet since becoming a caregiver (total *n* = 129–263) [34, 51, 53]. In one of these studies, higher caregiver burden was associated with an increased likelihood of caregivers reporting a negative change to their diet since becoming a caregiver [51]. The results of these studies are detailed in Table 5.

## 4 Discussion

Quantitative research on the dietary quality and intake of cancer caregivers remains largely inconclusive. Many studies included in this review reported on measures of dietary intake (e.g., frequency or amount consumed) or adherence to dietary recommendations and patterns, with mixed results. Estimates reported within these studies often resembled those reported for the general adult population [65, 66], with difficulties attributing these outcomes specifically to the caregiving role due to study design limitations, such as the lack of longitudinal data and control groups. Despite this, there is some evidence—primarily from retrospective self‐reported data—that caregiving may result in negative changes to dietary quality and intake [31, 34, 45, 46, 49, 51, 53].

There are several factors that may contribute to suboptimal dietary quality and intake among cancer caregivers. For example, caregiving involves both time and monetary costs [4, 5], which may result in reduced food intake or increased reliance on convenience foods that are often nutritionally imbalanced (e.g., containing high saturated fat and sodium) [67, 68]. Additionally, caregivers report prioritizing the needs of their loved one over themselves, serving as a barrier to them seeking and accepting support for their own health and wellbeing [69, 70]. This barrier may contribute to the relatively low level of support seeking [71] and high degree of unmet needs [72] among caregivers, particularly for those living in rural areas. While not specific to diet, these findings imply that dietetic support services may be under‐utilized by cancer caregivers. Due to the centralization of cancer services, many caregivers living in rural areas also need to travel into major cities to accompany their loved one during treatment, which can impede access to healthy foods due to barriers such as limited time, cost, stress, and fatigue [19]. Further, few interventions with group‐based nutrition education and cooking programs for people affected by cancer include family and friends [73].

To date, research on the impact of caring for someone with cancer on dietary quality and intake has relied predominantly on retrospective self‐reported data [34, 39, 45, 49, 51, 53]. Only one study identified in this review examined prospective changes in dietary intake over time and compared these changes between participants whose partner did or did not receive a cancer diagnosis during the study period; however, this study only measured fruit and sugar‐sweetened beverage intake [31]. Further, most studies that assessed the impact of caregiving on dietary quality or intake have assessed changes during early stages of the care continuum. For example, changes in the dietary intake of caregivers were measured when patients were hospitalized or undergoing treatment, although the direction of these changes varied [34, 49, 51, 53] or were not described [39]. While several other longitudinal and intervention studies have assessed intake in caregivers across multiple timepoints, follow‐up periods were often short (≤ 12 months) [32, 33, 40, 42]. Therefore, it is unknown how the diets of caregivers are impacted across different stages of the care continuum, from diagnosis through to treatment, survivorship, end‐of‐life care, and bereavement. In addition, few studies measured variables relevant to caregiving responsibilities and burden, to assess how the nature and intensity of caregiving influences dietary outcomes. To inform supportive care services, future research should monitor dietary intake patterns in individuals both before and after they become caregivers (e.g., Ezendam et al. [31]), and explore how these patterns change throughout treatment and beyond, and in relation to the nature and intensity of the caregiving role.

While the findings from this review provide insights into the diets of cancer caregivers, there were limitations in the measurement tools used to assess and interpret dietary outcomes. For example, variability in dietary outcomes measured across the studies reduced our capacity to synthesize the findings and assess the adequacy of caregivers' dietary quality and intake. In total, 21 different measurement tools were used, resulting in more than 30 outcomes (e.g., energy intake, fruit intake, diet quality), which were often reported using different units (e.g., cups per day, serves per day, pieces per day). Consistency of outcome measures and comparability with dietary recommendations is therefore needed to advance understanding in this field.

When selecting dietary assessment tools for future studies, it is important to use validated methods that are appropriate for the research aims and target population [74]. Validated 24‐h dietary recall tools, such as the ASA24 [54], were used across several studies to capture recent dietary intake estimates, typically over multiple random, non‐consecutive days as per recommended practice [37, 40, 42]. Some studies also used validated dietary screeners to assess intake over an extended period, such as the past month [33, 48, 49, 52]. Although screeners focus on specific nutrients and foods, and are also less accurate compared to 24‐h recalls, they are more suited to capturing habitual dietary intake [26]. Notably, many studies in this review used study‐specific tools containing broad questions such as, “How often do you eat a balanced diet including fruits and vegetables?” [30]. These questions may be interpreted differently depending on factors such as ethnicity and health literacy [75, 76]. As such, there appeared to be discrepancies between dietary intake estimates derived from 24‐h recalls and those derived from short questionnaires. For example, studies that used the ASA24 found that caregivers had low to moderate diet quality scores on average [37, 42], whereas studies that relied on single questions found that most (72%–77%) caregivers reported typically consuming a healthy diet [30, 47]. Relying on single items can be a limitation given the complexity of the diet, with previous research showing that short dietary screeners may be less valid and reliable for estimating dietary intake compared to more comprehensive methods like 24‐h recalls [59]. Discrepancies between study findings therefore underline the need for validated tools to accurately assess dietary quality and intake.

In this review, only 41% of studies examined overall patterns of dietary intake [30, 34, 37, 39, 42, 43, 45, 47, 51, 53], and 36% measured dietary quality or intake with reference to individual or population‐based guidelines [32, 34, 37, 38, 39, 41, 42, 52]. Considering that nutrients and foods are not consumed in isolation, it is the cumulative and interactive effects of multiple diet components that likely predict health outcomes [77]. When one component of the diet changes, it is usually compensated by another, reiterating the need to focus on overall dietary patterns [77]. Due to variation in the nutritional needs of adults based on factors such as age, sex, and physical activity levels, it is difficult to draw inferences around the state of caregivers' diets without explicit reference to dietary recommendations in studies. Few studies also measured contextual factors such as geographical remoteness, socioeconomic disadvantage, and food insecurity, which have been identified as strong predictors of diet‐related behaviors [78, 79]. Thus, future studies should focus on overall patterns of dietary intake, including diet quality, in relation to the individual needs and contexts of cancer caregivers.

### 4.1 Implications

**FIGURE 2:**
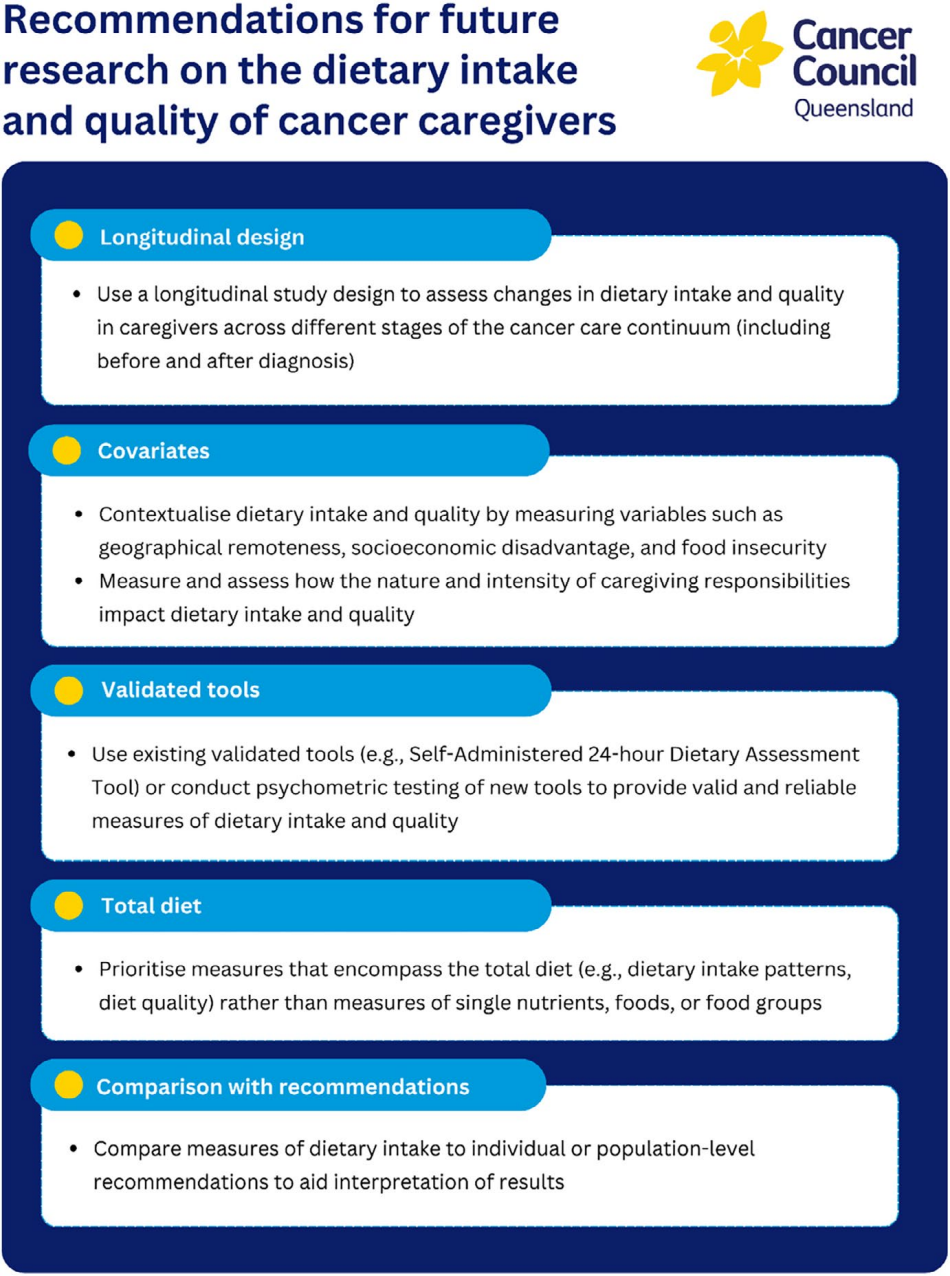
Summary of recommendations for future research examining the impacts of cancer caregiving on dietary quality and intake.

This research has implications for health professionals and researchers in optimizing supportive care for cancer caregivers. For health professionals, it may be relevant to investigate the dietary behaviors of caregivers and provide dietary information and support, particularly surrounding fruit and vegetable intake and overall diet quality, which can be compromised in this population group. Interventions might be particularly important during the early treatment phase and periods of hospitalization when caring responsibilities and burden are likely heightened. However, further research is needed to address limitations in existing studies to better understand the impacts of caregiving on dietary quality and intake. Recommendations for future research discussed above are outlined in Figure 2.

### 4.2 Limitations

There are several limitations that must be considered in the interpretation of these findings. Due to heterogeneity in the outcome measures, it was not feasible to conduct a meta‐analysis on dietary outcomes. Although most studies met at least three of the five criteria on the MMAT, the quality of the evidence overall was limited by low sample representativeness, mainly due to small sample sizes (median *n* = 72 [range = 21–672]) and convenience sampling. Hence, the findings from this review are predominantly applicable to white, educated, and married caregivers living in high‐income countries. The findings from this review are also reliant on self‐reported data, which are subject to social desirability and recall bias, with no studies using alternative methods such as photo‐assisted dietary assessments and grocery store purchase data [80]. While the aim of this review was to understand dietary quality and intake among cancer caregivers, studies focused predominantly on nutrients at risk of excess consumption (e.g., saturated fat, sodium), rather than those that might indicate a nutritional deficiency (e.g., iron, calcium). Additionally, data extraction and synthesis for this review were conducted by a single author, which may increase risks of bias and error compared to having two authors [81]. However, a second author reviewed the results to ensure their accuracy and completeness.

## 5 Conclusions

It is evident from this review that caregiving may negatively impact dietary quality and intake among cancer caregivers. However, results are largely inconclusive due to variation in dietary assessment methods and outcome measures used. Key recommendations for future research include: (i) prioritizing the use of validated tools that measure overall diet quality or dietary intake patterns with reference to dietary recommendations; (ii) monitoring the intake of individuals prospectively to understand the impacts of caregiving on dietary outcomes over time, and (iii) exploring the broader contextual factors that contribute to changes in dietary quality and intake among diverse groups of cancer caregivers.

## Author Contributions

**Susannah K. Ayre:** conceptualization (supporting), formal analysis (supporting), investigation (equal), methodology (lead), project administration (equal), supervision (supporting), writing – original draft (lead), writing – review and editing (equal). **Katelyn E. Collins:** formal analysis (lead), investigation (equal), writing – original draft (equal), writing – review and editing (equal). **Xanthia E. Bourdaniotis:** investigation (supporting), writing – review and editing (equal). **Grace L. Rose:** writing – original draft (equal), writing – review and editing (equal). **Gosia Boardman:** investigation (equal), writing – review and editing (supporting). **Constantina Depaune:** investigation (equal), writing – review and editing (supporting). **Belinda C. Goodwin:** conceptualization (supporting), supervision (supporting), writing – review and editing (lead). **Elizabeth A. Johnston:** conceptualization (lead), investigation (supporting), methodology (supporting), project administration (equal), supervision (lead), writing – review and editing (lead).

## Ethics Statement

This article reviewed existing literature. It did not involve human or non‐human (animal) participants, material, or data. Therefore, obtaining ethical approval was not required.

## Conflicts of Interest

The authors declare no conflicts of interest.

## Supporting information


Data S1:

## Data Availability

The data included in the systematic review are available within this article, complete with references.
