# Supplementary material for: Dietary Quality and Intake of Cancer Caregivers: A Systematic Review of Quantitative Studies and Recommendations for Future Research
Source: Cancer Med. 2025 Feb 10;14(3):e70668. doi: 10.1002/cam4.70668 (PMC11808751; doi:10.1002/cam4.70668)
Supplement: Supplementary file 1 — Data S1: [file CAM4-14-e70668-s001.docx]

**Supplementary Material 1:** Full search strategy used for five electronic databases.

| **Database** | **#** | **Search algorithm^a,b,c^** |
| --- | --- | --- |
| PubMed | 1 | cancer[Title] OR oncology[Title] OR tumo*[Title] OR neoplasm[Title] OR malignan*[Title] OR melanoma[Title] OR “Neoplasms”[Mesh] |
|  | 2 | caregiv*[Title/Abstract] OR carer*[Title/Abstract] OR caring[Title/Abstract] OR family[Title/Abstract] OR partner*[Title/Abstract] OR spous*[Title/Abstract] OR friend*[Title/Abstract] OR “Caregivers”[Mesh] |
|  | 3 | diet*[Title/Abstract] OR nutri*[Title/Abstract] OR food*[Title/Abstract] OR eating[Title/Abstract] OR intake*[Title/Abstract] OR consumption[Title/Abstract] OR fruit*[Title/Abstract] OR vegetable*[Title/Abstract] OR “Diet”[Mesh] OR “Eating”[MeSH Terms] OR “Diet Therapy”[MeSH] OR “nutritionists”[MESH] |
|  | 4 | 1. AND (2) AND (3) |
| Embase | 1 | cancer:ti OR oncology:ti OR tumo*:ti OR neoplasm:ti OR malignan*:ti OR melanoma:ti OR ‘malignant neoplasm’/exp |
|  | 2 | caregiv*:ab,ti OR carer*:ab,ti OR caring:ab,ti OR family:ab,ti OR partner*:ab,ti OR spous*:ab,ti OR friend*:ab,ti OR ‘caregiver’/exp |
|  | 3 | diet*:ab,ti OR nutri*:ab,ti OR food*:ab,ti OR eating:ab,ti OR intake*:ab,ti OR consumption:ab,ti OR fruit*:ab,ti OR vegetable*:ab,ti OR ‘diet’/exp OR ‘food intake’/exp OR ‘diet therapy’/exp OR ‘dietitian’/exp OR ‘nutritional counseling’/exp |
|  | 4 | (1) AND (2) AND (3) |
| CINAHL | 1 | TI cancer OR TI oncology OR TI tumo* OR TI neoplasm OR TI malignan* OR TI melanoma OR (MH “Neoplasms+”) |
|  | 2 | TI caregiv* OR TI carer* OR TI caring OR TI family OR TI partner* OR TI spous* OR TI friend* OR AB caregiv* OR AB carer* OR AB caring OR AB family OR AB partner* OR AB spous* OR AB friend* OR MM “Caregivers” |
|  | 3 | TI diet* OR TI nutri* OR TI food* OR TI eating OR TI intake* OR TI consumption OR TI fruit* OR TI vegetable* OR AB diet* OR AB nutri* OR AB food* OR AB eating OR AB intake* OR AB consumption OR AB fruit* OR AB vegetable* OR MH “Diet+” OR MM “Eating” OR MM “Dietitians” OR MM “Nutritionists” OR MH “Diet Therapy+” OR MH “Nutrition Services+” |
|  | 4 | (1) AND (2) AND (3) |
| Web of Science | 1 | TI=(cancer OR oncology OR tumo* OR neoplasm OR malignan* OR melanoma) |
|  | 2 | TI=(caregiv* OR carer* OR caring OR family OR partner* OR spous* OR friend*) OR AB=(caregiv* OR carer* OR caring OR family OR partner* OR spous* OR friend*) |
|  | 3 | TI=(diet* OR nutri* OR food* OR eating OR intake* OR consumption OR fruit* OR vegetable*) OR AB=(diet* OR nutri* OR food* OR eating OR intake* OR consumption OR fruit* OR vegetable*) |
|  | 4 | (1) AND (2) AND (3) |
| APA PsycINFO | 1 | TI cancer OR TI oncology OR TI tumo* OR TI neoplasm OR TI malignan* OR TI melanoma OR DE “Neoplasms” OR DE “Benign Neoplasms” OR DE “Breast Neoplasms” OR DE “Endocrine Neoplasms” OR DE “Leukemias” OR DE “Melanoma” OR DE “Metastasis” OR DE “Nervous System Neoplasms” OR DE “Terminal Cancer” |
|  | 2 | TI caregiv* OR TI carer* OR TI caring OR TI family OR TI partner* OR TI spous* OR TI friend* OR AB caregiv* OR AB carer* OR AB caring OR AB family OR AB partner* OR AB spous* OR AB friend* OR MM “Caregivers” |
|  | 3 | TI diet* OR TI nutri* OR TI food* OR TI eating OR TI intake* OR TI consumption OR TI fruit* OR TI vegetable* OR AB diet* OR AB nutri* OR AB food* OR AB eating OR AB intake* OR AB consumption OR AB fruit* OR AB vegetable* OR DE “Nutrition” OR DE “Beverages (Nonalcoholic)” OR DE “Calories” OR DE “Carbohydrates” OR DE “Diets” OR DE “Food” OR DE “Fast Food” OR DE “Eating Behavior” OR DE “Emotional Eating” OR DE “Healthy Eating” OR DE “Food Intake” OR DE “Dietary Treatment” |
|  | 4 | (1) AND (2) AND (3) |

^a^ **Search fields:** title (TI, ti), abstract (AB, ab), subject headings (DE, exp, Mesh, MH, MM).

^b^ **Search operators:** * = truncated term, + = exploded subject heading.

^c^ Searches were filtered to include articles published from 2013 onwards.
